# Humoral response and safety of the third booster dose of BNT162b2 mRNA COVID-19 vaccine in patients with multiple sclerosis treated with ocrelizumab or fingolimod

**DOI:** 10.1007/s00415-022-11296-4

**Published:** 2022-07-26

**Authors:** Rocco Capuano, Manuela Altieri, Miriana Conte, Alvino Bisecco, Alessandro d’Ambrosio, Giovanna Donnarumma, Elena Grimaldi, Nicola Coppola, Nicola Medici, Massimiliano Galdiero, Gioacchino Tedeschi, Antonio Gallo

**Affiliations:** 1 Department of Advanced Medical and Surgical Sciences (DAMSS), University of Campania “Luigi Vanvitelli”, Naples, Italy; 2 Department of Medical Sciences, Neurology Unit, AOU San Giovanni and Ruggi, Salerno, Italy; 3 Department of Experimental Medicine, University of Campania “Luigi Vanvitelli”, Naples, Italy; 4 Department of Mental Health and Public Medicine, University of Campania “Luigi Vanvitelli”, Naples, Italy; 5 Department of Precision Medicine, University of Campania “Luigi Vanvitelli”, Naples, Italy

**Keywords:** Booster dose, BNT162b2 mRNA vaccine, Ocrelizumab, Fingolimod, COVID-19

## Abstract

**Background:**

The assessment of the safety and the humoral response to a third booster dose of the BNT162b2 mRNA COVID-19 vaccine is relevant in patients with Multiple Sclerosis (pwMS) treated with Ocrelizumab (OCR) or Fingolimod (FNG).

**Methods:**

Serum samples were collected from Healthy controls (HCs) and pwMS treated with OCR or FNG at the following time-points: before the first of two vaccine doses (T0); 8 (T1), 16 (T2), 24 (T3) weeks after the first dose; within 8 weeks before (T0b) and after (T1b) the booster dose. IgG antibodies to SARS-CoV-2 trimeric spike protein (Anti-TSP IgG) were quantified and expressed as binding antibody units (BAU)/mL.

**Results:**

40 HCs, 28 pwMS on OCR and 19 on FNG were included. At T0b 12 (42.9%) pwMS on OCR and 6 (31.6%) on FNG were still positive while, at T1b 16 (57.14%) pwMS on OCR and 16 (84.2%) on FNG, passed the threshold of positivity. The increase of Anti-TSP IgG levels at T1b was higher for: (i) HCs with respect to OCR (*p* < 0.001) and FNG (*p* = 0.032) groups; (ii) pwMS on FNG compared with pwMS on OCR (*p* < 0.001). No socio-demographic, clinical or laboratory variables were able to predict the anti-TSP IgG increase between T0b and T1b. Neither clinical relapses nor severe adverse events were reported in pwMS after each dose of vaccine.

**Conclusions:**

The third booster dose of BNT162b2 mRNA vaccine to OCR- and FNG-treated pwMS revives the humoral response, independently of any clinical variable, and manifests a good safety and tolerability profile.

## Introduction

Despite the high efficacy of two doses of mRNA vaccines against SARS-CoV-2 [1, 2], a waning of the humoral immune response in healthy subjects was observed over 6 months, with a rise in the infection rate in fully vaccinated subjects after this time window [3]. Accordingly, National and International health organizations recommended a third booster dose of vaccine in most countries all over the world with a favorable impact on the risk of severe COVID-19 in healthy individuals [4].

The assessment of the safety and efficacy of the third booster dose is particularly relevant in patients with Multiple Sclerosis (pwMS) under treatment with high efficacy (HE) disease-modifying therapies (DMTs), which are known to strongly impact on the immune system.

Indeed, even if most pwMS were able to mount similarly to their healthy peers [1, 2] an efficient [5–7] and persistent (up to six months) [8, 9] humoral response after 2 doses of mRNA COVID-19 vaccine, a relevant percentage of those treated with two HE-DMTs such as Ocrelizumab (OCR) and Fingolimod (FNG) showed a blunted humoral response [7, 10, 11].

A third/booster mRNA vaccine dose could be, therefore, of paramount importance for boosting immune system and achieve more efficient protection against COVID-19 in these sub-populations of pwMS.

To date, there is a lack of data on the humoral response to the third vaccine dose against SARS-CoV-2 in pwMS under OCR and FNG, with preliminary evidence suggesting that the booster dose might provide a little but significant increase of IgG titers against the spike protein [12, 13].

Therefore, the aim of the present study was to investigate (i) the safety and the humoral response to the third booster dose of BNT162b2 mRNA COVID-19 vaccine in pwMS on OCR/FNG, comparing it with age- and sex-matched healthy controls (HCs), (ii) the relationship between longitudinal humoral response and routine clinical and immunological data in the studied population, and (iii) COVID-19 outcome in the enrolled MS population/sample vaccinated with 3 vaccine doses.

## Methods

This is an observational prospective study conducted at the Multiple Sclerosis Center of the I Neurologic Clinic of the University of Campania “Luigi Vanvitelli”.

We collected serum samples from HCs and pwMS on OCR/FNG at the following scheduled time points with respect to the first cycle (2 doses, 21 days apart) of BNT162b2 mRNA COVID-19 vaccine: before the first dose (T0; baseline), and 8 (T1), 16 (T2), 24 (T3) weeks after the first dose.

Two additional time-points were set to study the humoral response to the third booster dose of vaccine: within 8 weeks before the booster dose (T0b) and within 8 weeks after the booster dose (T1b).

Moreover, until March 31, 2022, for those participants who developed COVID-19 after the booster dose, we collected clinical and serological data (Tcov) within 4 weeks from the positive nasopharyngeal swab.

Exclusion criteria were: (i) age < 18 years; (ii) history of COVID-19 anytime before the third booster dose of vaccine, (iii) positive anti-spike SARS-CoV-2 IgG antibodies at T0, (iv) administration of corticosteroids within the month before the first vaccination cycle or before the booster dose, (v) relevant comorbidities potentially impacting on the immune system.

As previously reported [6, 8, 10, 11], all sera were stored at − 20 °C and tested at the Virology Lab of our University Hospital for the detection of IgG titers against SARS-CoV-2 trimeric spike protein (anti-TSP IgG), using the LIAISON® SARS-CoV-2 TrimericS-IgG assay (DiaSorin-S.p.A.) [14].

The Anti-TSP IgG titres were expressed as binding antibody units (BAU), with 33.8 BAU/mL as the negative/positive cut-off value [15].

The local Ethics Committee approved the study that was performed in accordance with the principles of the Helsinki Declaration.

### Statistical analysis

All statistical analyses were performed using SPSS, version 25.0. Data distribution was assessed using the Shapiro–Wilk test, due to the non-normality of distributions, a logarithmic transformation was performed for anti-TSP IgG values.

The comparison of clinical and demographic variables between pwMS subgroups (OCR vs FNG) was performed with chi-square (*χ*^2^) and Analysis of Variance (ANOVA), as appropriate.

Comparisons between groups of subjects that were positive at Anti-TSP IgG test were performed by the Fisher Exact Test.

To evaluate the effect of time and group of participants, and their interaction effect on levels of anti-TSP IgG, a multivariate analysis of variance (MANOVA) was performed, with anti-TSP at T0, T1, T2, T3, T0b and T1b as dependent variables, and the group of participants (pwMS on OCR, pwMS on FNG and HCs) as an independent variable.

To explore the effect of the booster dose, a univariate analysis of covariance (ANCOVA) was performed, with the percent difference of anti-TSP IgG between T0b and T1b (calculated with the following formula: (T1b − T0b) × 100) as a dependent variable, and a group of participants as an independent variable, controlling for the time elapsed between the first vaccine cycle and the booster dose. The magnitude of the effect size of MANOVA and ANCOVA was evaluated by calculating the partial eta squared (${\eta }_{\rho }^{2}$); the values 0.01, 0.06, and 0.014 were indicative of small, medium and large effect sizes, respectively [16].

Multiple regression analyses were performed to assess predictors of change in levels of anti-TSP IgG between T0b and T1b in the pwMS groups. The following predictors were added in the models: sex, age, and time elapsed between the first vaccine cycle and the booster dose, plus the time elapsed since the last infusion and the CD20 cells/mcL before the booster dose for the OCR group, or, alternatively, time on FNG, disease duration and absolute lymphocyte count (ALC) for the FNG group.

To rule out the occurrence of type 1 error, a Bonferroni correction for multiple comparisons was applied.

## Results

Among 162 pwMS treated at our MS Center and participating in an ongoing anti-SARS-CoV-2 serologic monitoring, we selected those on OCR (*n* = 28) and FNG (*n* = 19) who did not fulfil exclusion criteria. As a control group, we selected 40 age- and sex-matched HCs enrolled in a serologic surveillance program on COVID-19 at our Clinic.

All subjects received three vaccine doses of BNT162b2. Due to the vaccination schedule, serum samples at T3 were used also as T0b for 12 pwMS on OCR and 5 on FNG.

Socio-demographic and clinical characteristics of HCs and pwMS are reported in Table 1.

**Table 1:** Socio-demographic and clinical characteristics of HCs and pwMS

	HCs (40)	pwMS on OCR (28)	pwMS on FNG (19)	*p*
Age [years] mean (SD)	42.6 (12.8)	42.3 (9.4)	45.8 (13.5)	0.75
Female sex *n* (%)	23 (57.5)	13 (46.4)	8 (42.1)	0.52
Disease duration [months] mean, median (SD; IQR)	–	133.7, 138.2 (75.9; 61.7–186.9)	149.1, 127.2 (107.5;71.9–195.4)	0.82
EDSS–median (IQR)	–	4 (1.5–5.5)	2.5 (1.5–4)	0.38
Treatment duration [months]–mean, median (SD; IQR)	–	29.7, 32.1 (10.1; 22–336.2)	61.6, 72.7 (38.2; 17.9–88.7)	0.01
Time elapsed between first vaccine dose and booster dose [months]–mean, median (SD; IQR)	10, 10 (0.6; 9.5–10.3)	7.2, 6.9 (9.5; 6.4–7.6)	7.4, 7 (1.1; 6.5–7.9)	< 0.001* < 0.001** 0.47***
Time elapsed between last OCR infusion and first full vaccination cycle [months] mean, median (SD; IQR)	–	5.31, 4.3 (2.4; 3.3–7.5)	–	–
Time elapsed between last OCR infusion and booster dose [months] mean, median (SD; IQR)	–	4.8, 4.9 (0.7; 4.4–5.3)	–	–
Total CD20 lymphocyte within 30 days before first full vaccination cycle [cells/mcL] mean, median (SD; IQR)	–	25.9, 4.5 (69.4; 0–14.5)^a^	–	–
Total CD20 lymphocyte within 30 days before booster dose [cells/mcL] mean, median (SD; IQR)	–	9.8, 1.5 (15.1; 0–18)^a^	–	–
Serum IgG within 30 days before first full vaccination cycle [mg/dL] mean, median (SD; IQR)	–	863.1, 834.5^b^ (212; 686–1039)	–	–
Serum IgG within 30 days before booster dose [mg/dL] mean, median (SD; IQR)		816, 793.5 (191; 703–973)		
Total lymphocyte within 30 days before first full vaccination cycle [cells/mcL] mean, median (SD; IQR)	–	–	848, 772 (486; 560–1177)	–
Total lymphocyte within 30 days before booster dose [cells/mcL] mean, median (SD; IQR)	–	–	862, 750 (432; 490–1180)	–

*HCs* healthy controls, *pwMS* people with multiple sclerosis; *OCR* ocrelizumab, *FNG* fingolimod; *SD* standard deviation; *IQR* interquartile range; *EDSS* Expanded Disability Status Scores; *Anti-TSP IgG* anti-trimeric spike protein specific immunoglobulin G; *BAU/mL* binding arbitrary unit per ml; *NS* not significant

*Comparison between HCs and pwMS on OCR

**Comparison between HCs and pwMS on FNG

*** Comparison between pwMS on OCR and pwMS on FNG

^a^Normal range values 90–660 cell/mcL

^b^Normal range values 700–1600 mg/dL

Among the socio-demographic and clinical characteristics, HCs underwent the booster dose significantly later than pwMS (*p* < 0.001) while the only difference between OCR and FNG groups was the longer treatment duration of pwMS on FNG (*p* = 0.01).

T0b samples were collected 28.8 (standard deviation [SD] 24.8; median 29, P25 12, P75 37) days before the booster dose, while T1b samples were collected 33.9 (SD 12.8; median 30, P25 29, P27 35) days after the booster dose, with no differences between groups (*p* = 0.8 and *p* = 0.6).

Qualitative analysis showed that all HCs mounted a positive (> 33.8 BAU/mL) humoral response at T1 and preserved it during the follow-up, until and after the booster dose (T0b and T1b). On the other hand, at T0b only 12 (42.9%) pwMS on OCR (*p* < 0.001 compared with HCs) and 6 (31.6%) on FNG (*p* < 0.001 compared with HCs) were positive. At T1b, after the booster dose, 16 (57.14%) pwMS on OCR and 16 (84.2%) on FNG, passed the threshold of positivity (Table 2).

**Table 2:** Anti-TSP IgG > 33.8 BAU/mL at different time-points

	HCs (40)	pwMS on OCR (28)	pwMS on FNG (19)	*p*
T1 (8 weeks after first vaccine dose) number (%)	40 (100)	18 (64.3)	10 (52.6)	< 0.001* < 0.001** 0.5***
T2 (16 weeks after first vaccine dose) number (%)	40 (100)	16 (57.1)	10 (52.6)	< 0.001* < 0.001** 0.7***
T3 (24 weeks after first vaccine dose) number (%)	40 (100)	12 (42.9)	6 (31.6)	< 0.001* < 0.001** 0.5***
T0b (within 8 weeks before booster dose)	40 (100)	12 (42.9)	6 (31.6)	< 0.001* < 0.001** 0.5***
T1b (within 8 weeks after booster dose)	40 (100)	16 (57.1)	16 (84.2)	< 0.001* 0.03** 0.06***

Comparisons were performed by means of the fisher exact test. Significant values are reported in bold

*HCs* healthy controls; *pwMS* people with multiple sclerosis; *OCR* ocrelizumab: *FNG* fingolimod; *Anti-TSP IgG* anti-trimeric spike protein specific immunoglobulin G; *BAU/mL* binding arbitrary unit per mL

*Comparison between HCs and pwMS on OCR

*Comparison between HCs and pwMS on FNG

*** Comparison between pwMS on OCR and pwMS on FNG

Quantitative analysis showed significant higher anti-TSP IgG titers in HCs compared with those of pwMS on OCR and on FNG at all time points, while no differences were found at all time points between pwMS on OCR and those on FNG (Table 3).

**Table 3:** Log-transformed values of Anti-TSP IgG levels (BAU/mL) at different time-points and neutralising antibodies at T2

	HC (40)	pwMS on OCR (28)	pwMS on FNG (19)	*p*
Serum Anti-TSP IgG titre before vaccination (T0)–mean (SD)	0.69 (0.05)	0.71 (0.1)	0.68 (0)	0.54
Serum Anti-TSP IgG titre 8 weeks after first vaccine cycle (T1)–mean (SD)	3.36 (0.32)	1.9 (0.95)	1.6 (0.52)	< 0.001* < 0.001** 0.32***
Serum Anti-TSP IgG titer 16 weeks after first vaccine cycle (T2)–mean (SD)	2.97 (0.3)	1.63 (0.79)	1.47 (0.46)	< 0.001* < 0.001** 0.98***
Serum Anti-TSP IgG titer 24 weeks after first vaccine cycle (T3)–mean (SD)	2.72 (0.3)	1.45 (0.69)	1.36 (0.4)	< 0.001* < 0.001** 1***
Serum Anti-TSP IgG titre within 8 weeks before booster dose (T0b)–mean (SD)	2.4 (0.33)	1.42 (0.68)	1.27 (0.47)	< 0.001* < 0.001** 0.89***
Serum Anti-TSP IgG titre within 8 weeks after booster dose (T1b)–mean (SD)	3.93 (0.26)	1.84 (0.96)	2.18 (0.72)	< 0.001* < 0.001** 0.26***

Comparisons were performed by means of the ANOVA with Bonferroni post-hoc analysis. Significant values are reported in bold

*Anti-TSP IgG* anti-trimeric spike protein specific immunoglobulin G; *BAU/mL* Binding Arbitrary Unit Per ml; *SD* standard deviation

*Comparison between HCs and pwMS on OCR

**Comparison between HCs and pwMS on FNG

***Comparison between pwMS on OCR and pwMS on FNG

**Fig. 1:**
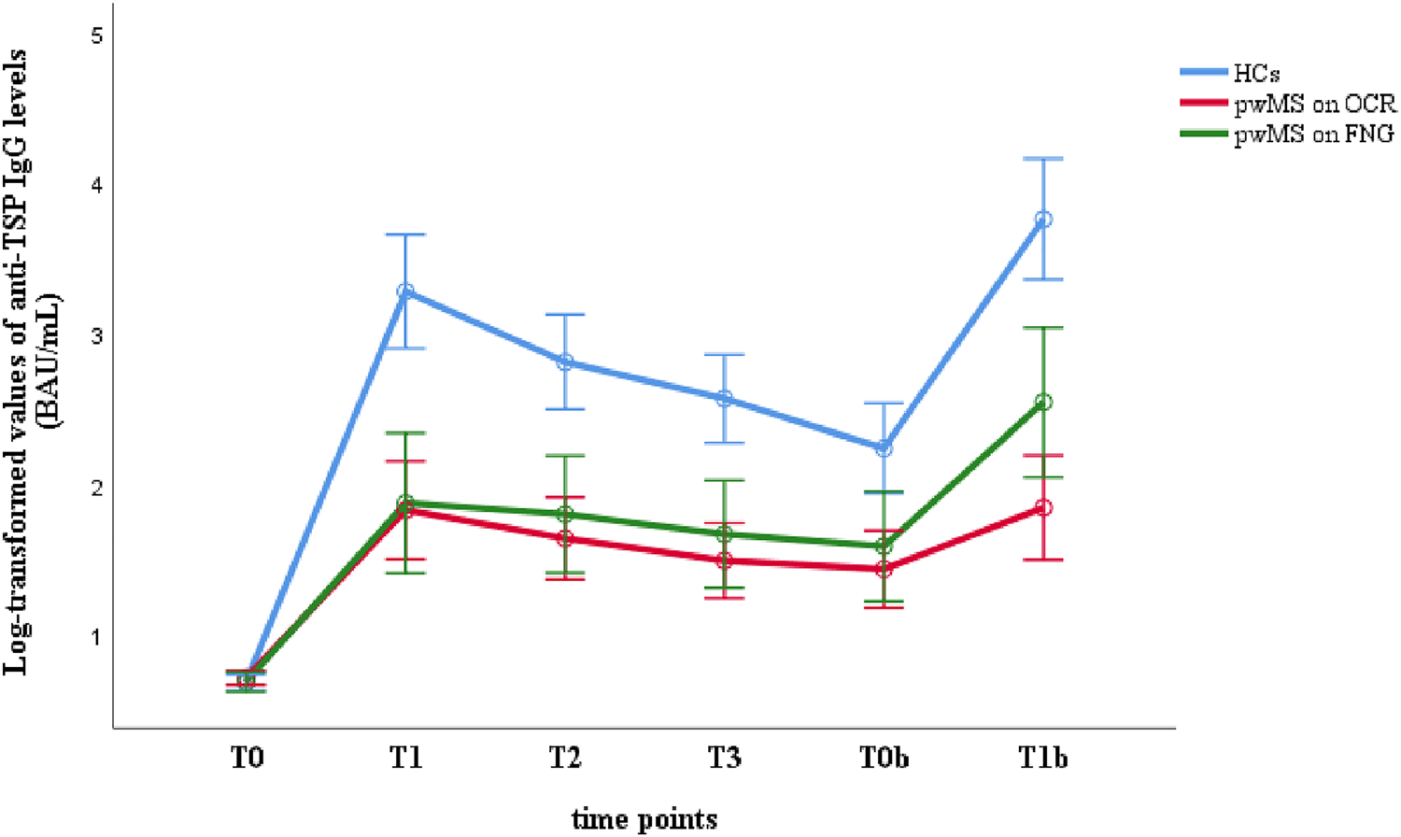
Log-transformed values of Anti-TSP IgG levels (BAU/mL) in pwMS and HCs. *anti-TSP IgG* anti-trimeric spike protein specific immunoglobulin G; *BAU/mL* binding arbitrary unit per mL; *HCs* healthy controls; *pwMS* people with multiple sclerosis; *OCR* ocrelizumab; *FNG* fingolimod; T0: baseline, T1: 8 weeks after the first dose, T2: 16 weeks after the first dose; T3: 24 weeks after the first dose; T0b: within 8 weeks before the booster dose; T1b: within 8 weeks after the booster dose

The repeated measures MANOVA revealed a significant main effect of time (Λ = 0.89, *F*(5,80) = 163.410; p < 0.001, *η*_*p*_^2^ = 0.911) and a significant interaction effect between time and group of participants (Λ = 0.154, *F*(10,162) = 24.808, *p* < 0.001, *η*_*p*_^2^ = 0.608), revealing that anti-TSP IgG levels in the HCs group were significantly higher than those of OCR and FNG groups at all time points after T0, with no differences between pwMS on OCR and those on FNG at any time-point (Fig. 1).

The ANCOVA aimed at evaluating possible differences between percentage increment of anti-TSP IgG levels between T0b and T1b revealed a significant effect on the group (*F*(2,87) = 16.979, *p* < 0.001, *η*_*p*_^2^ = 0.290). The HCs showed a mean increment of 150% (95% CI 128–172), whereas an increase of 44% (95% CI 21–67) and 99% (95% CI 75–123) was observed in the OCR and FNG groups, respectively. The post-hoc comparison with Bonferroni correction revealed that HCs showed a significant higher percentage increase of Anti-TSP IgG levels at T1b with respect to OCR (*p* < 0.001) and FNG (*p* = 0.032) groups; moreover, the increase in the pwMS on FNG was significantly higher than those in the OCR group (*p* < 0.001).

The multiple regression analysis to evaluate possible predictors of the percentage of increase of anti-TSP IgG levels between T0b and T1b did not reveal any significant predictor between socio-demographic (i.e. sex, age), clinical (i.e. time elapsed between the first vaccine cycle and the booster dose, the time elapsed since the last infusion before the booster dose for the OCR group, or time on FNG, disease duration for FNG group) and laboratory variables (i.e. CD20 cells/mcL for pwMS on OCR and ALC for those on FNG).

No serious or unexpected local and/or systemic side effects were observed in HCs and pwMS after the booster dose. Mild to moderate local and/or systemic adverse reactions (Adr) were reported after the booster vaccine dose in both groups. 44.7% of pwMS did not report Adr after the booster dose while, 53.2% reported pain at the injection site, 19.1% fever, 19.1% fatigue, 8.5% muscle or joint pain and 8.5% headache; frequencies of reported Adr were similar between pwMS treated with OCR or FNG. No clinical relapses neither EDSS worsening were reported in pwMS after the first two doses and the booster dose during the follow-up period.

Sixteen subjects got COVID-19 (4 HCs, 7 pwMS on OCR, 5 pwMS on FNG) 92 (standard deviation[SD] 42.1) days on average after the booster dose with no differences between groups (*p* = 0.4). All subjects presented a mild form of COVID-19, without the need for oxygenation, hospitalization or anti-viral and/monoclonal antibodies therapies. Mean log-transformed anti-TSP IgG levels after the booster vaccine dose and before COVID-19 were 3.86 (SD 0.42) BAU/mL for HCs, 2.19 (SD 1.04) for pwMS on OCR, and 2.52 (SD 0.62) for pwMS on FNG, whereas levels of anti-TSP IgG levels within 4 weeks from COVID-19 were 3.9 (SD 0.37) BAU/mL for HCs, 1.86 (SD 0.86) BAU/mL for pwMS on OCR and 3.06 (SD 0.76) for pwMS on FNG. No relapses or EDSS worsening were reported in the 4 weeks after COVID-19.

## Discussion

In the present study, we investigated the effects of a third booster dose of BNT162b2 mRNA SARS-CoV-2 vaccine in pwMS treated with OCR and FNG in terms of: (i) qualitative and quantitative humoral response, comparing it to age- and sex-matched HCs, (ii) safety; (iii) COVID-19 outcome in those patients contracting the infection (after the third booster dose). We also explored clinical and demographic factors predicting/influencing the humoral response to the booster dose.

As regards the first aim of the study, our data showed—as expected—that time significantly impacts anti-TSP IgG levels measured in pwMS on HE-DMTs. Indeed, while all HCs became positive after the first two doses and remained so until the time of the third/booster dose (T0b), only 42.9% of pwMS on OCR and 31.6% of those on FNG were still positive at T0b.

However, the third/booster vaccine dose was able to increase significantly anti–TSP IgG titers with 57.1% of pwMS on OCR and 84.2% of pwMS on FNG reaching the positivity threshold of the test.

These results, beyond confirming and expanding the evidences showing a weaker and shorter humoral response to the first two doses of BNT162b2 mRNA SARS-CoV-2 vaccine in pwMS treated with OCR and FNG [11, 17], demonstrate the efficacy of a third/booster dose in soliciting anti–TSP IgG seroconversion in these pwMS.

Moving to the quantitative analysis, the anti-TSP IgG titers of OCR- and FNG-treated pwMS were significantly lower at all time-points when compared with HCs; contrariwise, we did not find relevant differences—at any time-point—between pwMS on OCR and those on FNG.

Focusing on the third/booster dose, we observed a significant rise of anti-TSP IgG titers within 8 weeks before and after the booster dose in all 3 studied groups; once again, HCs showed a significant higher increase (150%) of anti-TSP IgG levels between T0b and T1b with respect to OCR (44%) and FNG (99%) groups. Interestingly, we observed that the increment of humoral response to the third vaccine dose in pwMS on FNG was significantly higher than that in the OCR group.

These results complement and expand two previous studies supporting the recommendation of a third vaccine dose in immunocompromised MS patients, such as those treated with OCR and FNG [12, 18].

The observed differences between pwMS on OCR and those on FNG might be explained on the basis of the different mechanisms of action of the two drugs on the immune system.

FNG determines a lower decrease/impairment of naïve B cells and plasmablasts and a higher decrease of memory B and T cells subsets [19]; the resulting imbalance of the different immune cells might bring to a strengthening of the humoral response instead of the B/T cellular response after the booster dose [20]. Contrariwise, OCR depletes all circulating B cells but spares CD20-negative plasma cells as well as T cells, therefore, despite an impaired and inadequate humoral response, a T-cell response is preserved, and similar to healthy pears, after SARS-CoV-2 mRNA vaccine [21–23].

Expanding the results of Konig et al. [12], we found no socio-demographic, clinical or laboratory parameters as predictors of the humoral response after the third booster dose. This evidence suggests that the effect of a third/booster vaccine dose on the immune system is strong and largely independent from patient-dependent variables, except for the assumption of drugs heavily impacting the immune system, such as OCR or FNG.

Moving to the second aim of our study, we did not observe any significant adverse event after the third/booster dose; mild/moderate reactions were commonly reported in HCs and pwMS, in line with previous studies, showing comparable rates in the general population and in pwMS [1]. We did not observe relapses in pwMS on OCR or FNG in the month after the booster dose, confirming vaccine safety as reported by previous studies on the first vaccine cycle [24]. The overall good safety and tolerability profile of a third mRNA vaccine dose further support its use in the MS population, including those immunocompromised by the therapy.

The third objective of our study was to understand COVID-19 outcomes in pwMS on OCR and FNG after 3 vaccine doses. We observed 12 (7 on OCR, 5 on FNG) mild cases of COVID-19 in pwMS, with no needing for oxygenation, hospitalization or anti-viral/monoclonal antibodies. Nevertheless, the small sample of subjects does not allow to draw any definitive conclusions on COVID-19 outcomes in pwMS on OCR or FNG.

Previous studies described the association between time since the last OCR infusion and FNG treatment duration with anti-TSP IgG titres after the first two mRNA vaccine doses [6, 7, 11]; we did not find any correlation between clinical or demographic factors and the humoral response after the third booster dose, in line with first reports [18]. This missing association might be due to the effect of the third booster dose, which might help counteract the immune system modifications due to drugs exposition; moreover, in pwMS on OCR, the effect of an additional infusion between the first vaccination cycle and the booster dose might impact on the effect of the humoral response. However, future studies with larger samples are needed to shed light on this issue.

This study is not exempt from limitations. First, all participants were vaccinated with the BNT162b2 mRNA SARS-CoV-2 vaccine; therefore, we were not able to assess the humoral response after other SARS-CoV-2 vaccines. Second, we did not assess the B and T cell response to the vaccine which is known to play an essential role in the immune response to infections and vaccines. Third, we only reported symptomatic cases of COVID-19 after the booster dose, this might underestimate COVID-19 cases. On the other hand, a strength of our study is the high number of serum samples obtained at different time points that made us able to promptly identify and exclude asymptomatic COVID-19 cases.

In conclusion, our results: (i) expand the growing evidence that pwMS on treatment with OCR and FNG are able to revive/raise their humoral response after a third/booster mRNA vaccine dose, independently of any demographic, clinical or laboratory metric/variable; (ii) confirm the good safety and tolerability profile of a third/booster dose of BNT162b2 mRNA SARS-CoV-2 vaccine, not only in terms of adverse events but also in terms of MS relapses; (iii) support the efficacy of 3 vaccine doses against severe COVID-19 course, even if in a very small/initial sample of pwMS.

## Data Availability

The data that support the findings of this study are available from the corresponding author, prof. Antonio Gallo, upon reasonable request.
